# In vitro investigation of *Datura innoxia* phytocompounds against *Mycobacterium tuberculosis* H37Ra strain in association with in silico studies

**DOI:** 10.1038/s41598-025-99053-1

**Published:** 2025-09-29

**Authors:** Sajjad Ahmed Khan, Muzafar Ahmad Rather, Ziyi Jia, Muhammad Umer Khan, Syed Mehmood Qadir, Hasan Ejaz, Muharib Alruwaili, Anthony D. Baughn, W. Thomas Shier, Muhammad Sheeraz Ahmad

**Affiliations:** 1 University Institute of Biochemistry and Biotechnology, PMAS-Arid Agriculture University Rawalpindi, Rawalpindi, 46300 Pakistan; 2 Department of Medicinal Chemistry, College of Pharmacy, University of Minnesota, Minneapolis, MN 55455 USA; 3 Department of Microbiology and Immunology, University of Minnesota Medical School, Minneapolis, MN 55455 USA; 4 Institute of Molecular Biology and Biotechnology, The University of Lahore, Lahore, 54000 Pakistan; 5 Department of Clinical Laboratory Sciences, College of Applied Medical Sciences, Jouf University, Sakaka, 72388 Saudi Arabia; 6 National Reference Laboratory for Tuberculosis, National TB Control Program, Islamabad, 44000 Pakistan

**Keywords:** *Datura innoxia*, *Mycobacterium tuberculosis* H37Ra, Minimum inhibitory concentration (MIC), MD simulations, Molecular docking, DFT analysis, Drug discovery, Microbiology

## Abstract

Tuberculosis (TB) is a recurrent and progressive bacterial disease caused by *Mycobacterium tuberculosis* (*Mtb*), posing a significant challenge globally due to its drug resistance. This study focuses on identifying natural phytocompounds from the plant *Datura innoxia* (leaves), which is well known for its biologically active metabolites. Initially, the current study employed in vitro analysis of 20 phytocompounds, revealing that the natural compound 9, o-vanillin, exhibited the best minimal inhibitory concentration (MIC) which was 12.5 µg/mL, and minimal bactericidal concentration (MBC) was 50 µg/mL, all other phytocompounds showing remarkable antitubercular activity against the *Mtb* H37Ra strain. The molecular docking and simulation also validated the strong affinity and stable binding interactions between compound 9 and target protein kinase. The pharmacokinetic analysis highlighted the suitable oral bioavailability and no significant CYP450 inhibition for the lead compound 9, reducing the risk for drug-drug interactions. Moreover, the density functional theory analysis of lead compound 9 demonstrated optimal molecular properties, further contributing to the chemical stability and reactivity. Therefore, these results suggest that *D. innoxia* contains the potent phytocompound o-vanillin, which possesses antitubercular activity and can potentially be used as a drug against TB. However, future studies will focus on in vivo validation and formulation development for clinical applications.

**Supplementary Information:**

The online version contains supplementary material available at 10.1038/s41598-025-99053-1.

## Introduction

Tuberculosis (TB) is a lethal, infectious disease. It is caused by the etiological agent *Mycobacterium tuberculosis* (*Mtb*)^1^. It is an aerobic bacillus that can infect any body part, especially the lungs^2^. According to a recent WHO report on TB, it is estimated that globally, 10.6 million people were infected with TB, with 1.3 million mortalities yearly^3^, with the highest burden in Asia and Africa^4^.

Back in ’90 s, there was a significant reduction in TB cases due to the introduction and discovery of antitubercular agents, such as isoniazid^5^, pyrazinamide, and rifampicin^6^. Isoniazid is considered the first-line antitubercular defense against TB, but unfortunately, the *Mtb* strains have shown resistance to isoniazid^7^. Similarly, rifampicin was also considered an important antitubercular agent, but some unavoidable effects have been reported against this key-player drug in the TB regimen^8^. *Mtb* resistance has also caused inefficiency in some of the 2nd line antitubercular drugs such as ethionamide, capreomycin, and kanamycin^9^. Thereby, with the misuse of these antitubercular drugs, there has been a significant rise in TB cases. Despite the accessibility of WHO-recommended directly observed treatment, short course (DOTS), drug resistance has become a significant barrier to TB eradication globally because of multidrug resistance (MDR) and extensive drug resistance (XDR)^10^. Although tremendous efforts have been made to discover targeted antitubercular drugs, most have failed to reach the clinical trial phase in the past six decades^11^. Therefore, the development of novel antitubercular drugs with different inhibitory targets that cause minimal or no side effects is urgently needed.

Notably, plants and humans exhibit significant associations^12^. Natural products retrieved from plants have always been essential sources of drug interventions^13^ due to their significant biological activities^14^. According to floristic studies, there are approximately 500,000 plant species, of which 120,000 contain biologically active metabolites that could serve as therapeutic agents^15^. Several secondary metabolites are greatly used as antitubercular agents, such as phenols, alkaloids, salicylates, coumarins, flavonoids, indoles, etc. The biomedical sciences domain has now emphasized the identification of therapeutic phytochemicals to overcome the challenges for the treatment of TB^16^.

The medicinal herb *Datura* belongs to the Solanaceae family and is commonly referred to as jimsonweed or thornapple^17^. A variety of *Datura* species are widely seeded on major continents, including Asia, Africa, Europe, America, and other subtropical and tropical regions^18^. This plant exhibits both toxic and medicinal properties, and various developing countries use this plant for herbal medicine preparations^19^. Several species of this plant possess multiple biological properties against various diseases, such as diabetes, cancer, asthma, and TB. Moreover, *Datura* species also exhibit anti-inflammatory, analgesic, antioxidant, cytotoxic, insecticidal, and neurological activities, as well as wound-healing properties^20^.

For the effective and economical identification of a potential phytocompound that could be used as an antitubercular drug, an in silico approach has proven to be a reliable method to be employed before actual synthesis^21^. It should be taken into account that the therapeutic interventions of phytocompounds are evaluated thoroughly, as there could be a chance of discovering more potent modes of action and targets as better candidates for drugs^19^.

Although natural phytocompounds have been identified for TB treatment, no such studies have systematically evaluated the phytocompounds of *D. innoxia* against the resistant *Mtb* H37Ra strain. Therefore, the current study was conducted on the phytocompounds present in the leaves of the *D. innoxia* plant to identify the antitubercular activity of these phytocompounds under controlled in vitro conditions and followed by an in silico analysis of the phytocompound by molecular docking, pharmacokinetic analysis, DFT calculations, and MD simulation to verify the potential of the compound to be used as a therapeutic drug against TB.

## Results

### Antitubercular activity

To develop novel antitubercular drugs, we analyzed 20 natural compounds from *D. innoxia* plant. The results illustrated in Table 1 show the MIC and MBC of these phytocompounds against the *Mtb* H37Ra strain. The antitubercular activity of the compounds **(9)**, **(7)**, and **(12)** showed the best inhibitory activity at 12.5 µg/mL, 50 µg/mL, and 50 µg/mL respectively and also have bactericidal activity with MBCs values of 50 µg/mL, 50 µg/mL and 200 µg/mL respectively. On the other hand, the maximum MIC value was observed for compound **(11)**, which was > 200 µg/mL, while all the other remaining compounds (**1)**,** (2)**,** (3)**,** (6)**,** (8)**,** (10)**,** (14)**,** (16)**,** (17)**,** (18)**,** (19)**, and **(20)** displayed moderately weak activity at approximately 100 µg/mL, and the following compounds **(4)**,** (5)**,** (13)**, and **(15)** showed extremely weak activity at 200 µg/mL against the *Mtb* H37Ra strain. The INH positive control, exhibited MIC and MBC values of 0.156 µg/mL. This suggests that out of all 20 phytocompounds, compound **(9)** showed strong momentous antitubercular activity under controlled in vitro conditions.

**Table 1:** Antitubercular activity of *D. innoxia* phytocompounds against *Mtb* H37Ra.

Compound code	Compounds name	MIC (µg/mL)	MBC (µg/mL)
**1**	Trans-ferulic acid	100	> 200
**2**	4-hydroxybenzoic acid	100	> 200
**3**	Methyl salicylate	100	> 200
**4**	(-)-scopolamine hydrobromide	200	N.D.
**5**	(-)-scopolamine N-butyl bromide	200	> 200
**6**	p-coumaric acid	100	N.D.
**7**	Norharmane	50	50
**8**	anisodamine (7β-hydroxyhyoscyamine)	100	200
**9**	**o-vanillin**	**12.5**	**50**
**10**	Nicotinic acid	100	N.D.
**11**	Atropine	> 200	> 200
**12**	Piperine	50	200
**13**	Scopoletin	200	200
**14**	Methyl isonicotinate	100	200
**15**	Methyl isonicotinate N-oxide	200	> 200
**16**	d-Damascone	100	> 200
**17**	3-Indoleacetic acid	100	> 200
**18**	3-Methylindole	100	> 200
**19**	2-Aminonicotinic acid	100	> 200
**20**	2-hydroxy-3-methoxy benzoic acid	100	200
**21**	INH	0.156	0.156
**22**	DMSO	> 200	> 200

MIC = minimum inhibitory concentration, mbc = minimum bactericidal concentration. Isoniazid (INH) used as positive control. DMSO was used as a negative control. N.D = Not determined.

## Molecular docking

**Fig. 1:**
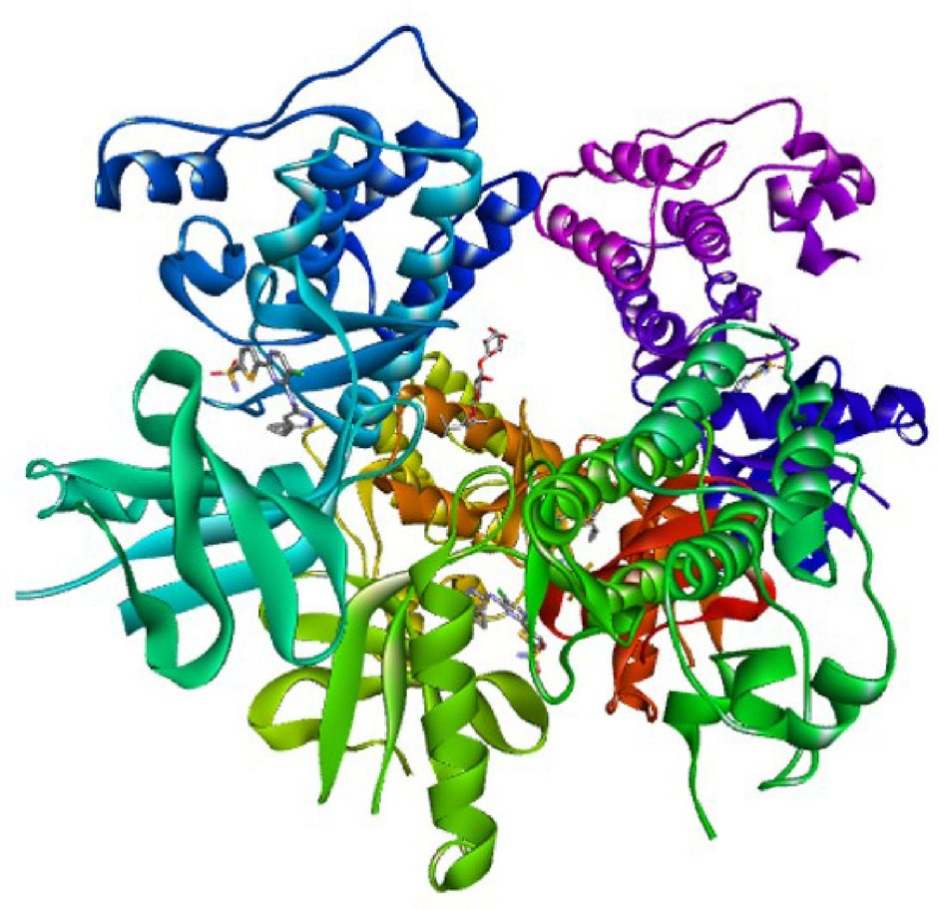
3-dimensional structure of the protein Pkn (PDB ID = 6B2Q), visualized on Discovery Studio Visualizer, (3D representation: PyMol Molecular Graphics system version 2.4.0).

**Fig. 2:**
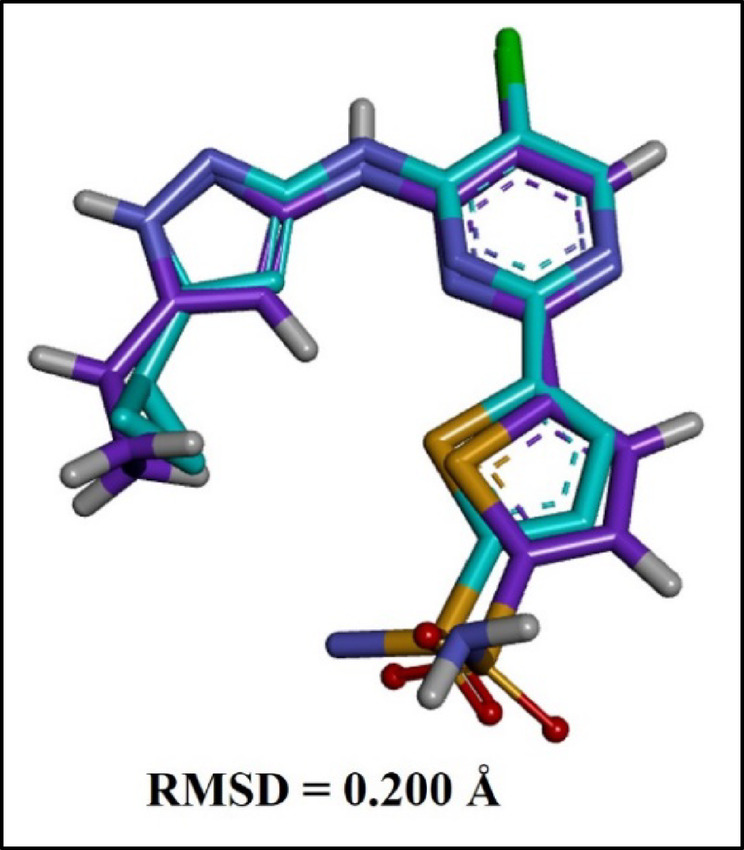
RMSD value of superimposed original CCL (PubChem CID: 50898364) (blue) and re-docked pose (purple).

The lead compound **(9)** was selected for in silico analysis based on strong evidence of its in vitro antitubercular activity. To further investigate the binding potential of the compound **(9)**, o-vanillin, with the targeted protein kinase (PDB ID: 6B2Q), involved dual inhibition of PknA and PknB, was analyzed as shown in Fig. 1. The binding affinity of compound **(9)**, reference (INH) drug, and CCL (PubChem CID: 50898364) was assessed based on the binding score and interactions with the target protein (PDB ID: 6B2Q). The docking procedure was verified by re-docking the CCL within the allosteric pocket of the protein by following the guidelines mentioned in the methodology section. The superimposed redocked structure shown in Fig. 2 validates the docking procedure as it exhibited an RMSD value of 0.200 Å^22^. The docking results of compound **(9)**, illustrated in Table 2, displayed a promising docking score of -6.836 kcal/mol, compared to the INH drug.

**Table 2:** 2D, 3D structures and SMILES along with the Docking score of CCL (PubChem CID: 50898364) and compound **(9)** o-vanillin, (PubChem CID: 8991)

Code	Name of the compound	2D Structure	3D Structure	Docking Score (kcal/mol)
**CCL**	5-[5-chloro-4-[(5-cyclopropyl-1 H-pyrazol-3-yl)amino]pyrimidin-2-yl]thiophene-2-sulfonamide			− 7.414
**INH**	Isoniazid			− 6.828
**9**	o-vanillin			− 6.836

**Fig. 3:**
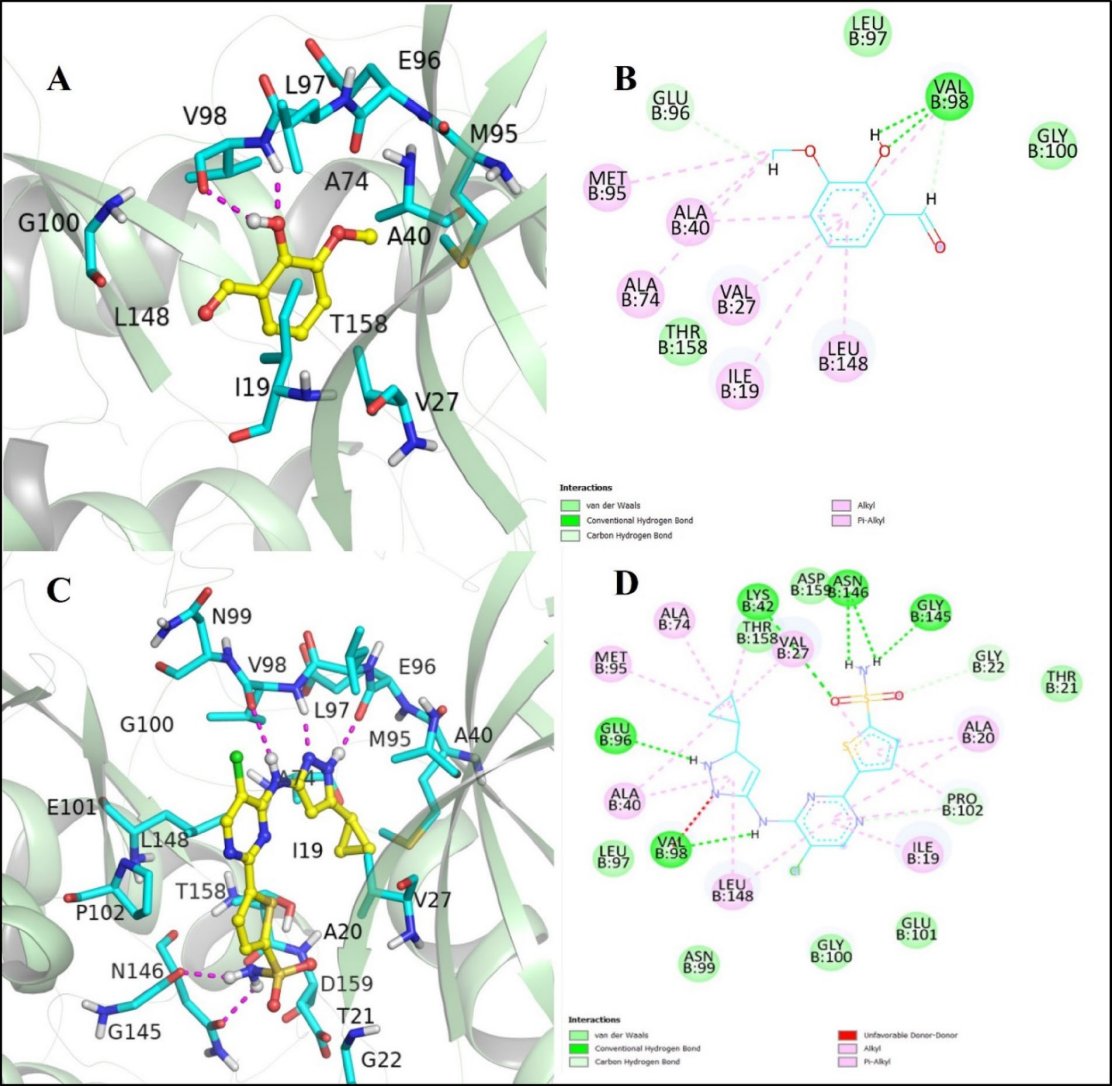
3D representation of the complex 6B2Q with the best inhibitory compound **(9)**, shown in (**A**), and CCL shown in (**C**) simultaneously, (**B**) and (**D**) show the 2D plot of the binding interactions between the compound **(9)** and CCL against protein Pkn (PDB ID: 6B2Q), respectively, (3D representation: PyMol Molecular Graphics system version 2.4.0; 2D representation: BIOVIA Discovery Studio Client 2021 version 21.1.0.20298).

The 2D and 3D interactions of compound **(9)**, viewed using Discovery Studio and PyMol (Fig. 3**)**, revealed the formation of a conventional hydrogen bond between VAL-98 amino acid residue and the hydroxy group located at the ortho position, relative to the aldehyde group. The Pi-alkyl interactions were formed between the benzoic moiety and ALA-40, ALA-74, VAL-27, ILE-19, and LEU-148 residues. Additionally, ALA-40 and ALA-74 also formed similar interactions along with MET-95, with the methoxy group present at the meta position to the aldehyde group. Simultaneously, LEU-97 and GLY-100 showed Van Dar Waal interactions, while THR-158 and GLU-96 exhibited a possibility of forming a carbon-hydrogen bond in the compound **(9)**.

In contrast, the CCL showed various conventional hydrogen bonds with the following amino-acid residues of the target protein: LYS-42, ASN-146, GLY-145, GLU-96, and VAL-98. It is noteworthy that the LEU-97 residue showed Van Der Waal’s interaction, and the THR-158 residue showed a carbon-hydrogen bond similar to the compound **(9)**. Moreover, CCL showed Pi-alkyl interactions with similar key amino acid residues (ALA-40, ALA-74, VAL-27, ILE-19, LEU-148, and MET-95) that are involved in the compound **(9)**. These interactions revealed a similar binding pattern and interactions with amino acid residues when compared to the interaction pattern of CCL.

Subsequently, the binding interactions of INH, compared to the compound **(9)**, revealed the formation of a favorable conventional hydrogen bond by the key residues i-e, VAL-98, THR-158, and GLU-96, with the carbonyl and hydroxyl groups of the INH drug. Additionally, non-covalent hydrophobic interactions were also formed by the key residues of the binding pocket, shown in **Supplementary Fig. 1**. Overall, the interactions highlighted that VAL-98, GLU-98, VAL-27, ALA-40, LEU-148, ILE-19, and THR-158 were considered as the major residues of the binding cavity involved in the inhibitory potential for therapeutic purposes.

### Pharmacokinetic analysis

The pharmacokinetic analysis of lead compound 9, compared with INH illustrated in Table 3, showed that both compounds fell within Lipinski’s cut-off range, referred to as the Lipinski rule of five (LRF), suggesting a suitable oral bioavailability for these compounds. The lipophilicity was measured as Log P_*o/w*_ (octanal-water partition coefficient), and solubility was measured as Log S for INH drug and compound 9, revealing that both compounds exhibited a suitable balance between hydrophilicity and hydrophobicity for a drug-like candidate. Subsequently, the drug-likeness of the compound 9 and INH drug indicated their potential use of the compound for therapeutic purposes. However, the medicinal chemistry showed that compound 9 and INH drug showed no pan-assay interference compounds (PAINS) alerts, indicating their reliability as a potent drug candidate. Whereas two Brenk alerts were observed in INH drug i-e., acyle hydrazine and hydrazine, and one Brenk alert was also possessed by the lead compound 9, i-e., aldehyde, indicating few structural liabilities. Moreover, the synthetic accessibility also highlighted the ease of synthesis for both compounds, particularly the lead compound 9. Additionally, the pharmacokinetic descriptors revealed high gastrointestinal (GI) absorption for the compound 9 and INH drug as well as highlighting that both of them were not a substrate p-glycoprotein (PGP), indicating a suitable therapeutic potential of compound **(9**), compared to the INH drug. Furthermore, compound 9 was also found to be not involved in any type of drug-drug interactions, similar to the INH drug.

**Table 3:** Pharmacokinetic analysis of the lead compound, compared to the INH drug, assessed through SwissADME. H-bond = hydrogen bond, TPSA = total polar surface area, PAINS = pan-assay interference compounds, CYP = cytochrome P450.

**Physiochemical properties**							
Code	Molecular weight (g/mol)	No. of heavy atoms	No. of rotatable bonds	H-bond acceptors	H-bond donors	TPSA (Å^2^)	
**9**	152.15	11	2	3	1	46.53	
**INH**	137.14	6	2	3	2	68.01	
**Lipophilicity**							
Code	Log *P*_o/w_	Log *P*_o/w_	Log *P*_o/w_	Log *P*_o/w_	Log *P*_o/w_	Consensus Log *P*_o/w_	
	(iLOGP)	(XLOGP3)	(WLOGP)	(MLOGP)	(SILICOS-IT)		
**9**	1.6	1.37	1.21	0.51	1.49	1.24	
**INH**	0.03	− 0.7	− 0.31	− 0.47	− 0.27	− 0.35	
**Water solubility and medicinal chemistry**							
Code	Log S	Log S	Log S	PAINS	Brenk	Synthetic accessibility	
	(ESOL)	(Ali)	(SILICOS-IT)				
**9**	− 1.92	− 1.95	− 1.88	0	1	1.16	
**INH**	− 0.56	− 0.25	− 1.64	0	2	1.24	
**Drug-likeness**							
Code	Lipinski	Egan	Veber	Bioavailability score			
**9**	Yes	Yes	Yes	0.55			
**INH**	Yes	Yes	Yes	0.55			
**Pharmacokinetics**							
Code	GI absorption	PGP substrate	CYP1A2	CYP2C19	CYP2C9	CYP2D6	CYP3A4
			Inhibitor	Inhibitor	Inhibitor	Inhibitor	Inhibitor
**9**	High	No	No	No	No	No	No
**INH**	High	No	No	No	No	No	No

## Density functional theory (DFT) calculations

**Fig. 4:**
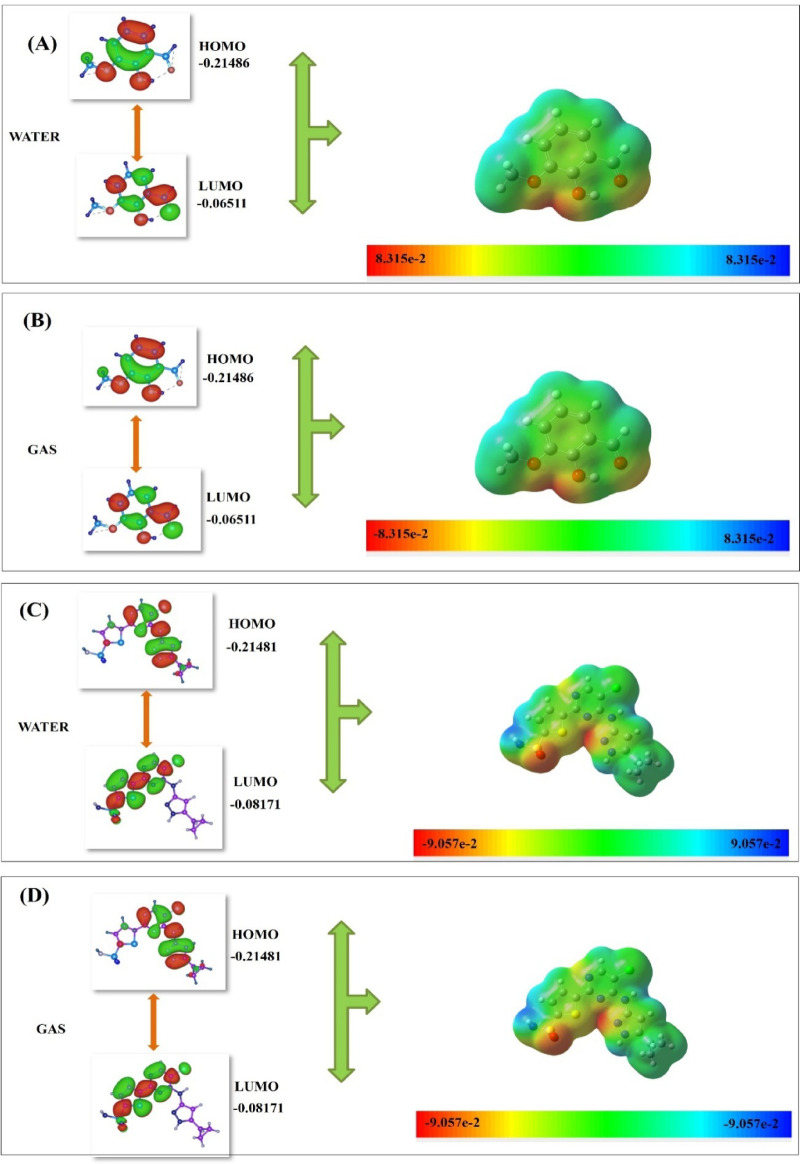
Optimized structural geometries of compound **(9)** and CCL, depicted in both solvent and gaseous phases (Figures **A** and **B** for compound **(9)**, and **C** and **D** for CCL), showed MEP. In the illustrations of MEP, the green areas represented neutral electrostatic potential, red indicated negative potential, and blue signified positive potential.

The DFT calculations for CCL and compound **(9)** were performed using Koopman’s theorem to compute the frontier molecular orbitals (FMOs) and global reactivity parameters^23^, illustrated in Table 4. The FMOs displayed in Fig. 4, highlighted the HOMO and LUMO energy, respectively, depicting electron donating potential and electron accepting ability of the molecules^24^, illustrated in Table 4. The difference between FMOs, represented as (ΔE_Gap_ = E_LUMO_ – E_HOMO_), is significant in analyzing the electronic transitions of the compounds. The comparative analysis of CCL and compound **(9)** showed that there is a slight variation in the energy gap (ΔE_Gap_), with compound **(9)** (0.14975 a.u.) being higher than CCL (0.1331 a.u.), indicating it to be more chemically stable than CCL^25^.

The result of global chemical reactivity descriptors illustrated in Table 4, showed that the ionization potential (*I*) of compound **(9)** and CCL was 5.84 eV, suggesting higher stability of both compounds^26^. On the other hand, the electron affinity (*A*) showed that a higher value of CCL made it more prone to accept the electron while a lower *A* of compound (9), suggested that it was more likely to be an electron donator^27^. The electronegativity (χ) outcomes of both compounds were almost similar, while the electrophilicity (ω) revealed that CCL was more reactive than the compound **(9)**. However, the chemical hardness (η) and softness (S) of the compounds revealed that both compounds had comparatively the same level of hardness, whereas compound **(9)** (0.49 eV) was significantly softer than CCL (0.55 eV). Moreover, the charge distribution in a molecule is visualized as MEP in Fig. 4 to determine the presence of binding sites for receptors or ligands^28^.

**Table 4:** Comparative DFT analysis, illustrating electronic, energetic, and global reactivity parameters for compound **(9)** and CCL.

Parameters for DFT analysis	CCL	Compound (9)
Dipole moment **(Debye)**	5.5290	4.9355
HOMO **(a.u.)**	− 0.21481	− 0.21486
LUMO **(a.u.)**	− 0.08171	− 0.06511
Energy Gap **(ΔE**_**Gap**_**)**	0.1331	0.14975
Ionization Potential **(*I*) (eV)**	5.84	5.44
Electron affinity **(eV)**	2.22	1.77
Electronegativity **χ (eV)**	4.03	3.81
Electrochemical potential **µ (eV)**	− 4.03	− 3.81
Hardness **η (eV)**	1.81	2.04
Softness **S (eV)**	0.55	0.49
Electrophilicity **ω (eV)**	4.49	3.56

### MD simulations

The docking studies showed protein-ligand binding interactions in a static condition while the MD simulation identified the complex’s structural conformation and motion in real time^29^. To observe the dynamic behavior of compound **(9)** and CCL with the target protein (PDB ID: 6B2Q), 100ns simulations were run, enabling us to scrutinize the fluctuation and adaptability within the allosteric cavity.

**Fig. 5:**
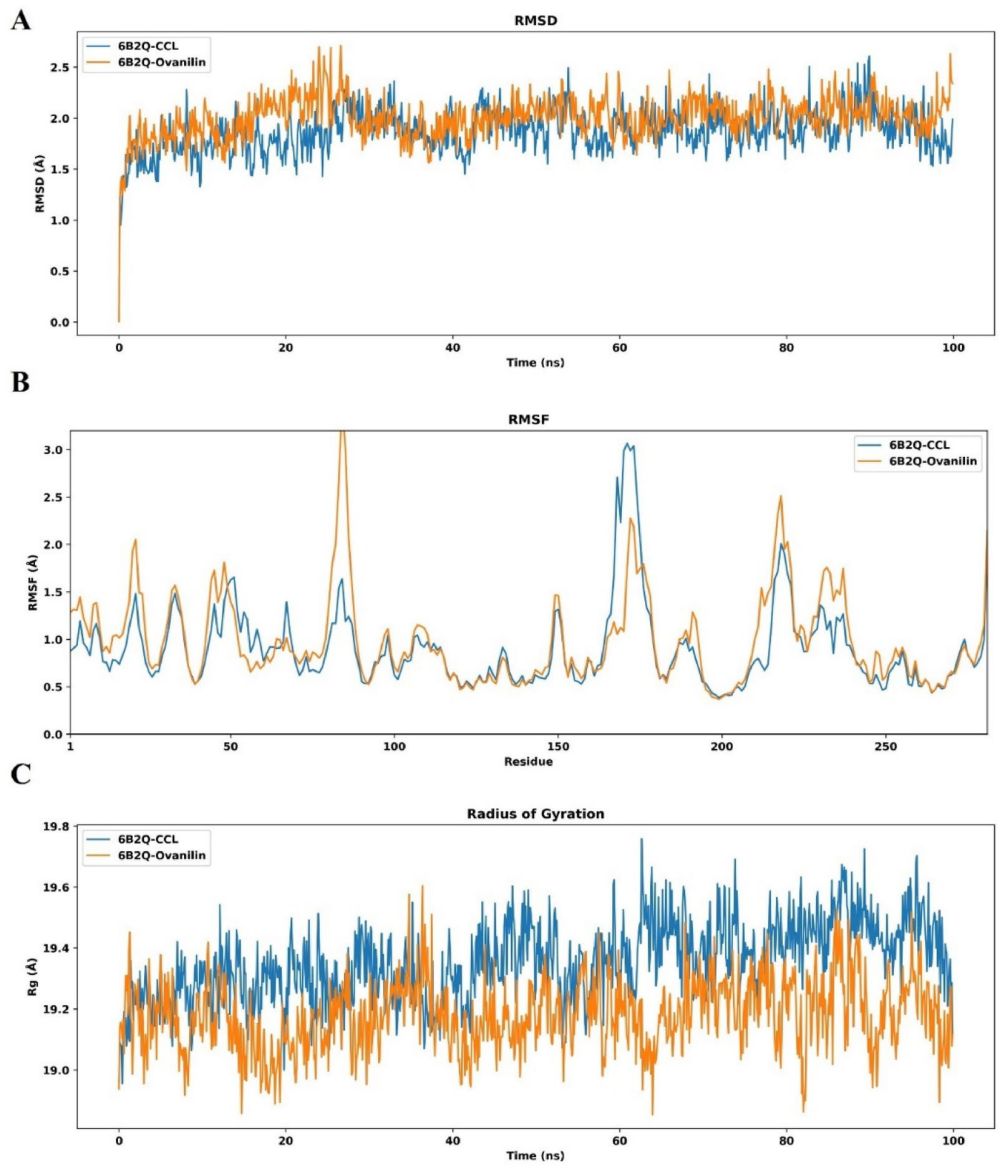
Graphical representation of the simulation. (**A**) RMSD fluctuation of CCL (blue) and compound **(9)** (orange), with time on the X-axis and RMSD on the Y-axis. Plot (**B**) portrayed the RMSF of CCL (blue) and compound **(9)** (orange), with time on the X-axis and RMSF on the Y-axis. Plot (**C**) displayed RGyr with time on the X-axis and RGyr on the Y-axis. RMSD = root mean square deviation, RMSF = root mean square fluctuation, RGyr = radius of gyration.

Figure 5**(A)** shows the RMSD values for the Cα atoms for both compounds. The RMSD plot revealed that compound **(9)** (represented in orange) and CCL (represented in blue) remained stable and steady throughout the 100 ns trajectory, characterized by minimal scattering in compound **(9)** for approximately 5 ns at the beginning of the simulations. It is noteworthy that the RMSD value of compound **(9)** maintains an RMSD below 3Å, indicating its stability within the protein’s binding site.

Figure 5**(B)**, displayed the RMSF analysis for the target protein, providing insights into the real-time behavior of its amino acid residues. The results showed the structural integrity of amino acids. However, a moderate level of fluctuation was observed in compound **(9)** (represented in orange) from GLY-80 to THR-90 residues of the protein but did not exceed more than 3 Å, indicating structural flexibility and conformational changes within the complex.

The overall compactness of the protein was determined by RGyr, shown in Fig. 5**(C)**. The results showed that the receptor in complex with compound **(9)** was more compact and consistent during the 100ns trajectory due to a lower level of fluctuations compared to CCL, which showed a slightly higher fluctuation moving towards the N-terminus of the protein, suggesting less compactness of the protein bound to CCL.

Moreover, the total number of hydrogen bond interactions between compound **(9)** and the target receptor, in comparison to the CCL docked complex, was also analyzed over 100ns simulation trajectory. The plot illustrated in Supplementary Fig. 2 revealed a dynamic binding pattern for CCL with minor fluctuations around 75 ns, indicating transient binding stability. Whereas, the hydrogen bond analysis of the lead compound 9 showed that hydrogen bond donors were more continuous than hydrogen bond acceptors, and mainly fluctuates between 6 and 9 over time. It was noteworthy that no complete loss of hydrogen bond was observed during the 100 ns trajectory with a pronounced fluctuation around 50 ns, indicating flexible and stable conformational changes within the docked complex.

## PCA-based FEL analysis

**Fig. 6:**
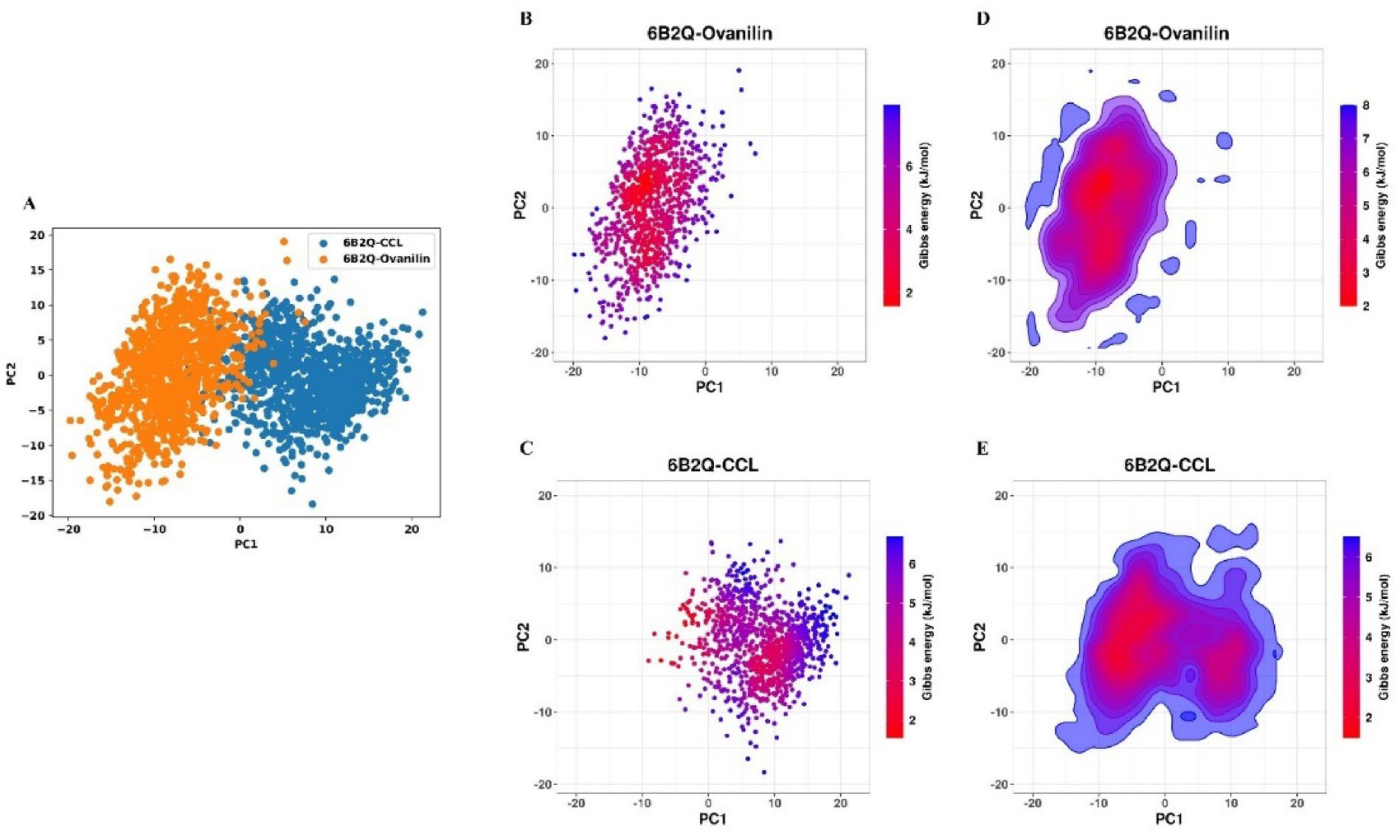
PCA of the compound **(9)** in comparison to the CCL. (**A**) Combined scattered plots for both complexes, (color-coded blue = CCL and orange = P-2). (**B**&**C**) individual scattered plots of CCL and compound **(9)** in complex with target protein 6B2Q, respectively. (**D**&**E**) plots colored with Gibb’s energy respectively.

The PCA investigated the conformation dynamics of the complexes during a 100ns simulation trajectory. The plots shown in Fig. 6, revealed that although both complexes showed variation in conformational dynamics. The docked complex with CCL showed a more scattered distribution, highlighting its higher conformational flexibility. Whereas, the lead compound **(9)** possessed more compact cluster, signifying its greater stability during 100ns simulation trajectory further enhancing its inhibitory efficacy against the target protein.

**Fig. 7:**
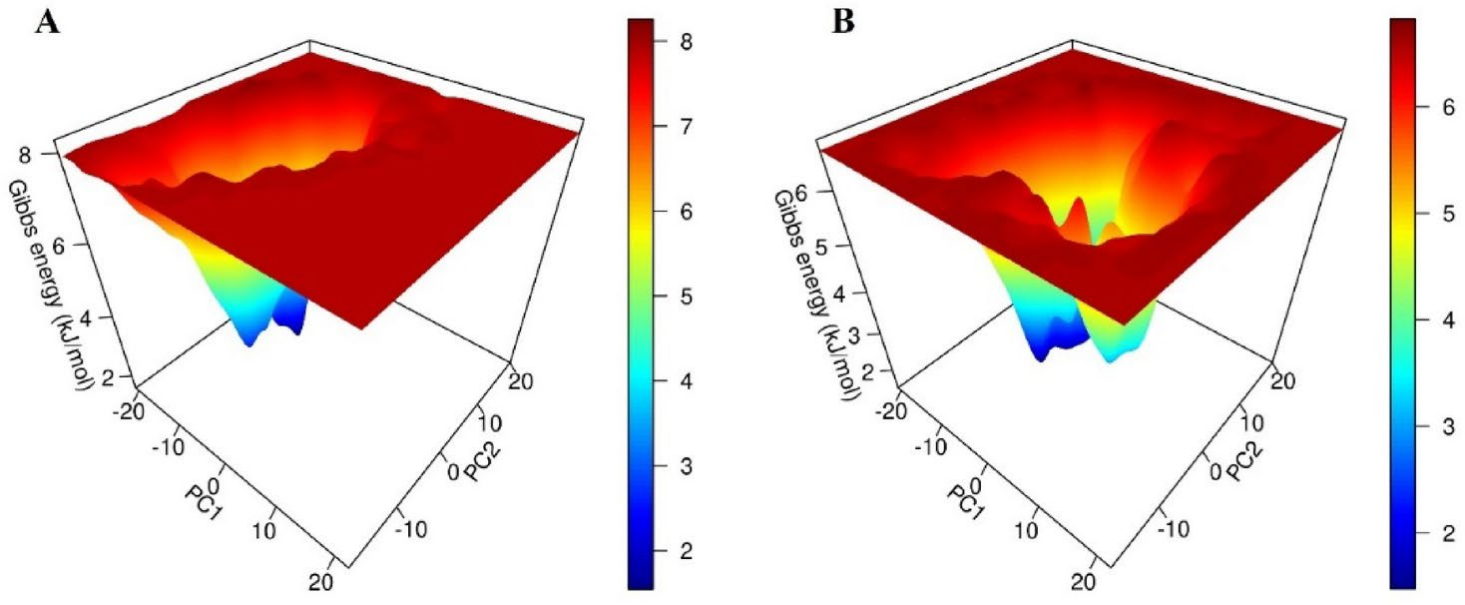
(**A** & **B**) represents the FEL of the CCL and compound (9) across PC1 and PC2. FEL = free energy landscape.

Moreover, the results of FEL **(**Fig. 7**)** showed a compact conformation of the ligand binding site in both complexes, indicated as Gibbs free energy across principal components. The plots depicted that the lead compound (**9**) exhibited a lower energy basin, indicating a more stable binding conformation with minimal energy fluctuation compared to CCL. These results suggested that the compound 9 remains stabilized within the binding pocket of target protein by minimizing the entropic effect, which could ultimately improve its efficacy as a therapeutic agent against drug resistant *Mtb*.

## Discussion

Tuberculosis is a lethal communicable disease, specifically caused by *Mtb*. The reemergence of TB over the past few decades due to drug resistance has posed a significant challenge affecting healthcare. This challenge has necessitated the swift identification and development of new antitubercular drugs^30^. The field of biological sciences has revolutionized by focusing on the treatment of multiple diseases through natural compounds. Researchers are currently concentrating on utilizing comprehensive in vitro and in silico approaches to explore natural phytocompounds as promising drug candidates. An experimental study conducted by Khan M.U. et al. investigated the efficacy of the natural compound quercetin and armepavine as a potential drug candidate against cystic fibrosis^31^. Similarly, another study employed in silico studies to search for novel natural compounds against neurological disorders^32^.

The current study aimed to identify the phytocompounds from the *D. innoxia* plant which is capable of producing biologically active metabolites against TB^20^. This research employed in vitro analysis on 20 biologically active phytocompounds of *D. innoxia* as antitubercular agents, as reported in the literature. The in vitro analysis revealed that compound 9 was capable of maximum inhibitory activity with a minimal inhibitory concentration of 12.5 µg/mL against the *Mtb* H37Ra strain, suggesting it is a remarkable phytocompound to be used as an antitubercular agent. The potential of compound 9 as an inhibitor was also confirmed by another study that demonstrated the in vitro efficacy of vanillin against *Leishmania tropica*^33^. Another experimental research conducted by Ravindran R. et al.^34^ also aligns with the current study, showing the inhibitory potential of conventional plants against *Mycobacterium smegmatis.* Similarly, one of the studies showed the MIC value of *Datura* species against the *Mtb* H37Rv strain, indicating the potential of biologically active phytocompounds to treat TB^35^.

Based on the in vitro inhibitory potential of lead compound 9 against *Mtb* H37Ra, docking studies were performed along with DFT analysis and MD simulations to gain further knowledge on the potential binding affinity and interactions of the compound against the receptor of this bacterium. A similar approach was also implemented in various studies for the identification of novel compounds against drug-resistant bacteria. This approach is further supported by research conducted on the natural compound propolis, employing both in vitro and in silico analysis, to combat anti-microbial drug resistance^19^.

The results of the molecular docking showed an acceptable binding affinity with the protein kinase (PDB ID: 6B2Q) in a static condition. The binding interactions of the lead compound 9 exhibited a strong H-bond with the VAL-98 and GLU-96 amino acid residue of the protein, along with Pi-alkyl and van der Waals interactions among key amino acid residues such as ALA-40, ALA-74, VAL-27, ILE-19, and LEU-148 involved in CCL. These interactions showed stable binding conformation of the lead compound within the binding pocket of the target protein, which was further validated by MD simulations. A study also confirmed the inhibitory potential of the target protein kinase B (PknB) as an attractive target of *Mtb* H37Ra for the identification and discovery of novel drug candidates^36^. Moreover, experimental research conducted to identify novel inhibitors revealed six compounds as potent drug candidates against a similar target, protein kinase^37^.

The pharmacokinetic analysis of the lead compound in comparison to the INH drug also provided essential details for the compound to assess its therapeutic potential as an antitubercular agent. The pharmacological results comply with the LRF, underscoring its favorable oral bioavailability. Followed by moderately balanced hydrophilic and hydrophobic profiles highlighting the membrane permeability and absorption of the lead compound 9 within physiological space. Moreover, the lead compound was also observed to be PGP negative, revealing that it is unlikely to be rapidly effluxed from the cells, similar to the INH drug. It is noteworthy that the high Log P value of lead compound 9 suggested its improved permeability, which correlates with its significant binding affinity with the target protein. Likewise, with the lack of CYP450 inhibition in INH drug, the lead compound 9 was also found unlikely to interfere with the metabolic enzymes, thereby reducing the risk for drug-drug interactions as well as altered drug metabolism. However, the aldehyde group of lead compound 9, highlighted as Brenk alert, suggested metabolic liability; simultaneously, this structural feature may also have contributed to its stronger binding interaction with the active site residues of the target protein. Therefore, further optimization of this functional group may enhance the stability of the compound while retaining its bioactivity for therapeutic purposes.

DFT calculations determined the molecular properties of the lead compound 9 in comparison to CCL. The HOMO and LUMO energy levels provided an insight into the donation and acceptance of electrons in the molecule, highlighting its significance for molecular interactions, specifically with protein-ligand binding. The results illustrated in Table 3 show that the significant properties of the compound 9 exhibited strong reactivity and stability. The energy gap is considered a key indicator of chemical stability and reactivity, suggesting greater chemical stability and suitable reactivity for the lead compound 9. This stability and reactivity of compound 9 also aligns with the docking and simulation studies underscoring strong and persistent interactions with key interacting residues (VAL-98, ALA-40, and GLU-96) throughout the simulation trajectory. Additionally, the stable RMSD value and strong hydrogen bonding during simulations also supported the stability and reactivity of the lead compound 9. Moreover, the global reactivity parameters, such as electronegativity and electrophilicity, also showed that CCL is more prone to non-specific interactions due to its higher reactivity, whereas a balanced reactivity of compound 9 contributed to selective binding to the target protein, as visualized in docking studies. Furthermore, higher ionization energy and lower electron affinity of lead compound 9 indicated enhanced stability within the binding pocket of the target protein. Subsequently, the formation of hydrogen bonds with VAL-98 residue and hydrophobic interactions with ALA-40 and LEU-148 also aligns with the molecular electrostatic potential visualized in the MEP analysis. Yernale et al. have also applied this approach to investigate novel compounds as antimicrobial and antioxidant agents. Further, the DFT calculations of natural compounds were also computed by Jayaraman M. et al.^38^, showing the molecular properties of the natural compound to be used as inhibitors against *Mtb* H37Ra. Therefore, by integrating DFT findings with molecular docking and simulations, the current study provides an in-depth understanding of the electronic properties of compound 9 contributing to its binding affinity and stability against the target protein, further underscoring its potential as a promising antitubercular agent.

Finally, the compound was subjected to MD simulations to determine the stability of the complex in real time. Other studies have also utilized this approach to observe the stability and conformational changes within the complex^39^. The results of the current investigation showed that in the phytocompound in complex with the target protein kinase (PDB ID: 6B2Q), the RMSD value remained within an acceptable range of 3 Å, and the RMSF value also remained within the limit of 3 Å, suggesting that fluctuations within the amino acid residues were responsible for the molecular motion, leading to functional modulation. The overall analysis showed that the complex remained steady and stable, showing flexibility during a 100ns simulation trajectory. These results suggested a more specific interaction pattern between ligand and receptor, which correlates to higher binding affinity and sustained inhibition of the target protein. Additionally, the RGyr quantifies the compactness of docked complex. The pronounced compactness in the lead compound 9 correlates with the stable RMSD and RMSF patterns, hence validating its potential as an effective inhibitor for therapeutic purposes. Furthermore, the stability and lower conformational changes in compound 9 docked with target protein underscore its efficacy for a promising drug like candidate for future drug development. Simultaneously, the hydrogen bond analysis also highlighted that compound 9 bind effectively with the receptor due to the formation of stable hydrogen bonds throughout simulation trajectory. This also correlates with the docking results, further supporting the therapeutic efficacy of compound 9 against resistant *Mtb* H37Ra strain. These results are further supported by a study conducted on the *Mtb* H37Rv strain on PknG in complex with phytocompounds, showing promising results in the in silico studies^40^. These findings offered a potent lead compound 9, o-vanillin, as an antitubercular agent for combating TB due to drug resistance.

## Conclusion

The emergence of drug-resistant *Mtb* poses a significant health challenge. From this perspective, the current study reflected that the phytocompound o-vanillin, retrieved from the plant *D. innoxia*, exhibited potential biological inhibitory activities, as confirmed by in vitro analysis. Moreover, in silico analysis showed valuable binding affinities and interactions while determining the molecular characteristics of o-vanillin as a suitable antitubercular drug candidate. However, in vivo analyses are yet to be performed to further validate the efficacy of this compound for safe delivery with minimal side effects against TB.

### Methodology

**Fig. 8:**
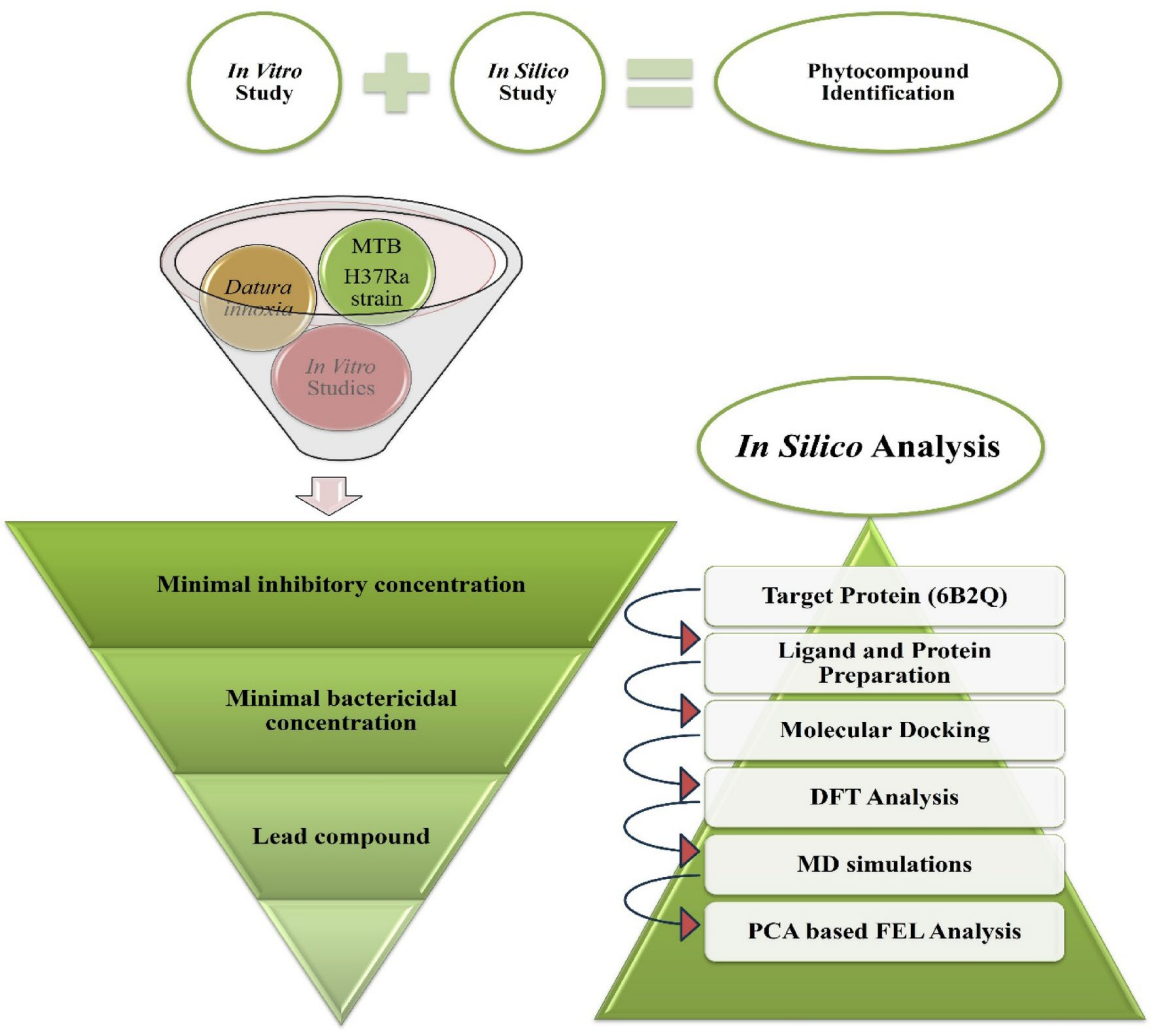
Approach used in the current study for identifying phytocompound against *Mtb* H37Ra.

The current study employed comprehensive in vitro and in silico methodology, displayed in Fig. 8 for the identification of promising phytocompounds from the *D. innoxia* plant.

## In vitro study

### Phytocompounds for bioassay

Initially, we explored the anti-tubercular properties of various medicinal plants and found that *Datura innoxia* was the most potent. Therefore, we selected twenty phytocomponents from *Datura innoxia* that have biological active in order to investigate its anti-tubercular potential. Twenty natural phytocompounds include trans-ferulic acid, 4-hydroxybenzoic acid, (-)-scopolamine hydrobromide, (-)-scopolamine N-butyl bromide, norharman, p-coumaric acid, anisodamine (7β-hydroxyhyoscyamine), o-vanillin, nicotinic acid, atropine, piperine, scopoletin, Methyl isonicotinate, Methyl isonicotinate N-oxide, d-Damascone, 3-Indoleacetic acid, 3-Methylindole, 2-Aminonicotinic acid, and 2-hydroxy-3-methoxybenzoic acid were purchased in pure form for antitubercular analysis in this study detail. The commercial sources and authentication numbers of the compounds were detailed in Supplementary Table 1.

### Compounds dissolution and dilution

Initially, 4 mg of each compound was dissolved per mL of dimethyl sulfoxide (DMSO), followed by two-fold serial dilutions, resulting in a concentration; 200 µg/mL to 0.39 µg/mL for each compound. INH positive control was tested at concentrations range from 0.625 µg/mL to 0.039 µg/mL.

### Microorganism strain

The *Mtb* strain utilized in the current study was the *Mycobacterium tuberculosis* H37Ra strain (*Mtb* H37Ra), to evaluate the antitubercular activity of phytocompounds.

### Growth conditions of bacteria

The *Mtb* H37Ra strain was grown in a sterile PETG media bottle containing 10 mL Middlebrook 7H9 broth supplemented with 10% (w/v) OADC (Difco, Becton Dickinson, USA), 0.05% (w/v) tyloxapol, and 0.2% (v/v) glycerol (Sigma Chemical Co.). The growth conditions were maintained at 37 °C with mild shaking at 100 rpm in a shaking incubator (Innova 4900, New Brunswick Scientific, USA) until *Mtb* H37Ra strain growth reached the log phase with an optical density (OD) of 0.1–0.3 at 600 nm. The culture was diluted to a final OD_600_ of ~ 0.01 for the antitubercular assay. Simultaneously, Middlebrook 7H10 agar media supplemented with 0.2% glycerol and 10% (w/v) OADC (Difco Becton Dickinson, USA) was prepared to measure the minimum bactericidal concentration (MBC) of *Mtb* H37Ra.

### Determination of minimum inhibitory concentration and minimum bactericidal concentration by microdilution method – MTT assay

3-[4,5-Dimethylthiazol-2-yl]-2,5-diphenyltetrazolium bromide (MTT) was used to determine the antitubercular activity of the phytocompounds against *Mtb* H37Ra, with a slight modification of the method according to Martin et al.^41^. All phytocompounds were diluted two-fold in DMSO and added 5µL in a 96-well microtiter plate and then 95µL of the *Mtb* H37Ra culture suspension (final OD_600_ = 0.01) was inoculated into each well, followed by incubation at 37 °C for one week. INH was used as a positive control, and DMSO was used as a negative control because it is not active against this bacterium. Simultaneously, the MBC was recorded as the lowest concentration that killed 99% of the colony-forming units (CFU) in the initial inoculum. For this purpose, 10µL was taken from each well, inoculated onto Middlebrook 7H10 agar plates, incubated at 37 °C, and read after 3 weeks. Then 10µL of MTT solution was poured into each 96-well plate containing the inoculum and incubated at 37 °C overnight. If violet-colored precipitation was observed visually, then 50µL of SDS-DMF formazan solubilization buffer was added, followed by incubation at 37 °C for another 3–4 h. A color change from yellow to violet indicates bacterial growth, and the MICs were interpreted accordingly^42^. The experiments were performed in triplicate and the MIC was recorded by visual observation.

## In silico analysis

### Target protein retrieval

Data from the Research Collaboratory for Structural Bioinformatics Protein Data Bank (RCSB PDB) (RCSB PDB: Homepage), accessed on September 23, 2024, were used to retrieve the crystal structure of protein kinase (Pkn), PDB ID: 6B2Q, which is majorly involved in signal transduction. The structure retrieved was in PDB format and contained four chains, namely A, B, C, and D, each having a sequence length of 317 amino acids without any mutation at a resolution of 2.88 Å^43^.

### Ligand retrieval

The lead compound was selected as the ligand for the in silico analysis. The phytocompound was retrieved in 2D and 3D structures from PubChem and sketched by using ChemDraw Professional 16.0 software (version: 16.0.1.4.77). The 3D structure was saved in the SDF format for docking purposes and the MOL format for DFT calculations.

### Ligand preparation

The Ligprep tool available on Schrödinger’s 2020-3 (Maestro Version 12.5.139) was utilized for ligand preparation of the co-crystal ligand (CCL) and lead compound for obtaining structural optimization. The Epik module was employed to generate 32 poses for each ligand, producing desalts and tautomers at pH 7.0 ± 2. Finally, each ligand was minimized under a force field of OPLS3e^44^.

### Protein preparation

The Protein Preparation Wizard tool, easily available on Schrodinger 2020-3 (Maestro Version 12.5.139) was employed to conduct the protein preparation of the target protein, PDB ID: 6B2Q. The missing loops and side chains were filled by the prime job, and the Epik module was employed to generate het states between pH = 7.0 ± 2, and water molecules beyond the diameter of 3 Å were deleted. Moreover, PROPKA was employed at pH = 7.0 to optimize the H-bond scaffolding. The steric hindrance was removed for further refinement and modification, and finally, the protein was minimized under the OPLS3e force field^45^.

### Glide grid generation

Receptor grid generation was done using the receptor grid generation panel in the Maestro glide program available on Schrodinger 2020-3 (Maestro Version 12.5.139) in the centroid of the working space of the CCL for the identification of suitable interactions between the receptor and the ligand^46,47^.

### Molecular docking

The molecular docking of lead compound and INH was done along with CCL on Maestro, Schrodinger 2020-3 (Maestro Version 12.5.139), with the Ligand Docking tool. The receptor grid file and the energy-minimized files of the ligands were imported^48^ and the docking was set to extra precision, flexible ligand sampling with the per residue scoring of 12Å of grid generation along with the calculation of RMSD to input ligand geometries. Finally, the 2D interactions of the docked complexes were viewed on Discovery Studio Visualizer v21.1.0.20298, and the 3D interactions were viewed on PyMol Molecular Graphic System version 2.4.0.

### Pharmacokinetic analysis

The pharmacokinetic analysis of the lead compound and INH drug was performed on SwissADME (SwissADME), an online tool, by applying the molecular fingerprinting technique. The string of canonical SMILES of the query molecule was provided as input data to evaluate the ADME across various descriptors, including physiochemical features, lipophilicity, solubility, pharmacokinetics, drug-likeness, and medicinal chemistry.

### DFT calculations

The three-dimensional geometries of the lead compound and CCL were designed and optimized by using GaussView (version 5.0.8). The CPCM model was chosen for the optimization and frequency calculations in the solvent and gaseous phases using the B3LYP method. The frontier molecular orbitals (FMOs) and global reactivity parameters were computed, along with a visualization of the molecular electrostatic potential (MEP) of the molecules.

### MD simulations

MD was performed on Desmond to determine the natural setting behavior and stability of the protein-ligand docked complex in real-time^29^. Initially, Maestro’s Protein Preparation Wizard was used to optimize and minimize the complex. The settings were done by the System Builder Tool, and the complexes were dissolved with the simple point-charge (SPC) solvent model in an orthorhombic box with dimensions of 10Åx10Åx10Å. The physiological conditions were mimicked by adding 0.15 M NaCl. The simulation trajectories were run under the OPLS4 force field at 300 K temperature and 1 atm pressure for NPT production. Nosé–Hoover chain coupling scheme and Martyna–Tuckerman–Klein chain coupling scheme, respectively, were used to control the temperature and pressure^49^. The trajectories of the docked complexes of o-vanillin and CCL were saved after every 100 ps during a 100 ns simulation. The stability and conformation were analyzed based on the root mean square deviation (RMSD) and root mean square fluctuation (RMSF) during the course of the simulation^50^. Lastly, Desmond trajectories were converted to XTC format for the computation of the radius of gyration using the gmx gyrate command of GROMACS^51^.

### Principal component analysis (PCA) – based free energy landscape study (FEL)

PCA is a multivariate statistical technique performed by computing the protein’s internal motion denoted by eigenvalues and eigenvectors. The covariance matrix for the C-alpha (Cα) coordinates was generated over a 100 ns simulation trajectory^52^. PCA was conducted in Python language via the scikit-learn library^53^. Moreover, FEL analysis was conducted on principal components (PC1 and PC2) to gain insight into conformational states relying on the energy distribution throughout the complex using GROMACS.

## Electronic supplementary material

Below is the link to the electronic supplementary material.

Supplementary Material 1

## Data Availability

All data generated or analyzed during this study are included in this published article.
