# Supplementary material for: In vitro investigation of Datura innoxia phytocompounds against Mycobacterium tuberculosis H37Ra strain in association with in silico studies
Source: Sci Rep. 2025 Sep 29;15:33454. doi: 10.1038/s41598-025-99053-1 (PMC12479965; doi:10.1038/s41598-025-99053-1)
Supplement: Supplementary file 1 — Supplementary Material 1 [file 41598_2025_99053_MOESM1_ESM.docx]

**Supplementary Data**

**Supplementary Table 1:** Test Compounds for Anti-TB Activity: Commercial Sources and Authentication Details

| **Test Compounds** | **Company, Item, and Lot Number** |
| --- | --- |
| trans-Ferulic acid | TCI America, H0267, Lot 7Y7JF-GH |
| 4-Hydroxybenzoic acid | Sigma-Aldrich Co., H20059-100G, Pcode 102540940 |
| (-)-Scopolamine hydrobromide | Sigma-Aldrich Co., S1875-1G, Pcode 1003522453 |
| (-)-Scopolamine N-butyl bromide | Sigma-Aldrich Co., S7882-1G, Lot BCCD7058 |
| Norharmane | Cayman Chemical Co., 20043, Batch 0488379-11 |
| p-Coumaric acid | Sigma-Aldrich Co., C9008-5G, Pcode 102555485 |
| Anisodamine (7β-hydroxyhyoscyamine) | Sigma-Aldrich Co., SML0252-10MG, Batch 0000161122 |
| o-Vanillin | TCI America, H0262, Lot MNL6G-DA |
| Nicotinic acid | Sigma-Aldrich Co., 72310-100G, Lot 0001389899 |
| Atropine | Cayman Chemical Co., 12008, Batch 0481334-32 |
| Piperine | Cayman Chemical Co., 11750, Batch 0462878-30 |
| Scopoletin | Cayman Chemical Co., 20042, Batch 0487716-14 |
| Methyl isonicotinate | Oakwood Chemical, 040095, Lot 040095P03X |
| Methyl isonicotinate N-oxide | Oakwood Chemical, 013166, Lot 013166J06G |
| d-Damascone | Chem-Impex Intl Inc., 34060, Lot 002344-0588 |
| 3-Indoleacetic acid | Sigma-Aldrich Co., I2886-5G, Lot SLCL0192 |
| 3-Methylindole | Sigma-Aldrich Co., M-2127, Lot 80H0728 |
| 2-Aminonicotinic acid | Acros Organics, 104160050, Lot A0345574 |
| 2-Hydroxy-3-methoxybenzoic acid | Sigma-Aldrich Co., 196495-25G, Pcode 1003510179 |


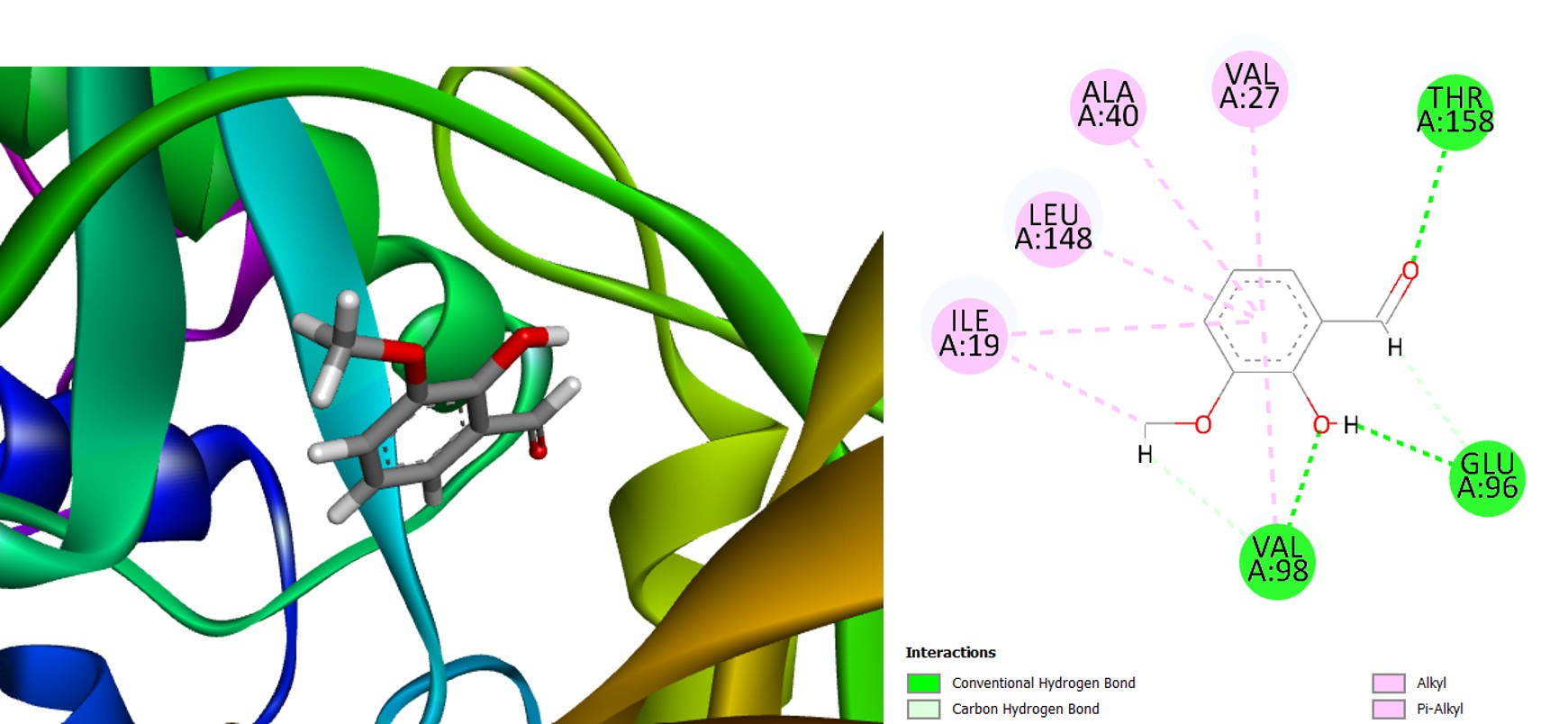


**Supplementary Fig 1**. Binding interaction of R* drug, isoniazid, against protein Pkn (PDB ID: 6B2Q), respectively.


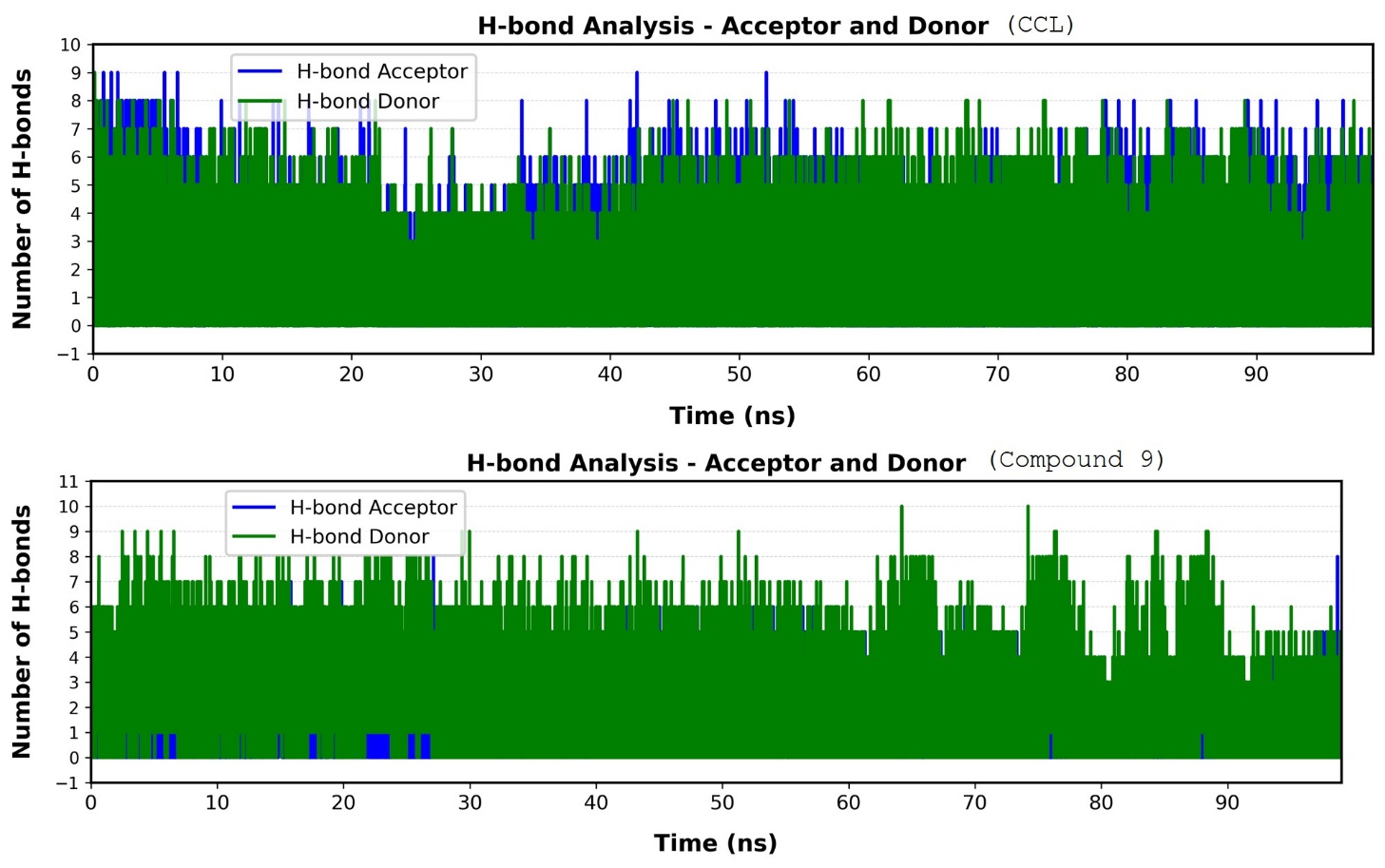


**Supplementary Fig 2**. Hydrogen bond interactions of CCL and compound 9, against protein Pkn (PDB ID: 6B2Q), respectively.
